# Towards standardization of training and practice of reconstructive microsurgery: an evidence-based recommendation for anastomosis thrombosis prophylaxis

**DOI:** 10.1007/s00238-018-1417-0

**Published:** 2018-04-09

**Authors:** Marie C. Kearns, Jill Baker, Simon Myers, Ali Ghanem

**Affiliations:** 1 Academic Plastic Surgery, Barts and the London School of Medicine and Dentistry, London, UK; 2 Canniesburn Plastic Surgery Unit, Glasgow Royal Infirmary, Glasgow, UK; 3 Department of Plastic Surgery, St John’s Hospital, Livingston, West Lothian UK; 4 Centre for Cutaneous Research, Blizard Institute - Barts and The London School of Medicine, 4 Newark St, London, E1 2AT UK

**Keywords:** Microsurgery, Free flap surgery, Thrombosis, Heparin, Aspirin, Dextran

## Abstract

**Background:**

Despite significant improvements in survival rates, free flap failures still occur even in experienced hands and are most commonly due to arterial or venous thrombosis. In the absence of an evidence-based guideline on the prevention of thrombosis, we reviewed the literature to assess the evidence base for commonly used interventions aimed at its prevention.

**Methods:**

A comprehensive literature search was performed using the following keywords “free flap” and microsurgery with “pre-operative screening,” “prevention of thrombosis,” “ketorolac,” “heparin,” “low molecular weight heparin,” “aspirin,” “dextran,” and “statins.”

**Results:**

Thirteen clinical studies were included in this review. No high-level evidence is available to support any perioperative or postoperative interventions aimed at reducing the risk of flap thrombosis.

**Conclusions:**

Higher level studies are needed to investigate the clinical use of antithrombotic medications in microsurgery; however, given the small failure rates in modern practice, these will need to be large multicenter trials in order to reach sufficient power.

Level of Evidence: Level III, risk/prognostic study.

## Introduction

Free flap reconstruction has seen vast improvements in survival rates over the past 30 years with success rates of 95–99% reported in the literature [1, 2]. These advances are attributed to refinements in microsurgical technique, improvements in instruments, and patient selection. Meticulous technique and an experienced microsurgeon are commonly regarded as the key to success in free flap reconstruction. However, even in the hands of experts, failures do occur due to arterial or venous thrombosis. Patient factors that have been associated with an increased risk for free flap failure include atherosclerosis, obesity, presence of chronic wounds, and previous radiotherapy [2, 3]. Free flap failure leads to delays to prolonged hospital stays, increased costs, delays in rehabilitation, and delays to adjuvant treatment for cancer patients. The surgeon is faced with planning an alternative method of reconstruction with possible challenges including a lack of recipient vessels, and the patient must deal with additional donor site morbidity and the psychological challenge of undertaking further surgery.

This article aims to review the current literature and propose an evidence-based approach to interventions aimed at minimizing the risks of thrombosis in free tissue transfer.

## Methods

Searches were made of Medline, Embase, and Cochrane Library using the following search terms; microsurgery and free flap with anticoagulant, heparin, low molecular weight heparin, aspirin, dextran, statins, prevention of thrombosis, ketorolac, and preoperative screening. Titles and abstracts were reviewed to select papers suitable for full text review, and references of relevant articles were manually searched for relevant publications. Searches were guided by the PRISMA statement and searches were independently completed by MK and JB. Risk of bias was assessed using the Cochrane Risk of Bias Tool. Citations were managed using Endnote Version 7.

### Search strategy

(microsurgery OR “free flap”) AND (anticoagulant OR heparin OR aspirin OR “low molecular weight heparin” OR LMWH OR dextran OR statins OR ketorolac OR “prevention of thrombosis” OR “pre-operative screening”)

Eligibility criteria (1) included patients 18 years of age or older, (2) included patients undergoing free flap reconstructive procedures, and (3) designed to assess the effect of a particular perioperative or postoperative intervention on the risk of pedicle thrombosis. Studies which did not include a comparative or control group, studies including less than 20 patients, and those with no English full text available were excluded. The primary outcomes were venous or arterial thrombosis, flap failure/survival, and return to theater.

## Results

The literature search returned 493 results. Thirteen studies were included in this review which combined included 4668 free flaps. These included 1 level 1 study, 1 level 2 study, and 11 level 3 studies [4–16]. Interventions evaluated included aspirin, heparin (topical, intraoperative bolus and postoperative prophylactic dosing or infusion), low molecular weight heparin (LMWH), dextran, PGE1, ketorolac, and topical recombinant human tissue factor pathway inhibitor (rhTFPI). Four studies compared groups who were treated with a combination of anticoagulants, and 3/13 studies assessing the impact of anticoagulation had a negative control group which received no anticoagulation. Two included studies were published by the same group including the same series of patients [6, 7]. One compares differences in thrombosis with an intraoperative bolus of heparin without detailing the differences in postoperative anticoagulation detailed in the other and vice versa.

The details of included studies are detailed in Table 1.

**Table 1:** Outline of included studies detailing level of evidence and main findings

Reference	Study type (level of evidence)	Population (*n*)	Intervention	Results	Weaknesses
Khouri et al. 2001 [4]	Multicenter RCT (level 1)	Free flap reconstruction (*n* = 622)	1. rhTFPI 0.05 mg/ml	Thrombosis rates NS	No negative control group
			2. rhTFPI 0.15 mg/ml	Hematoma lower in low-dose rhTFPI group, − 3 versus 8% in high-dose group, and 9% in heparin group (*p* = 0.04)	
			3. 100 U/ml heparinized saline		
Kroll et al. 1995 [5]	Retrospective cohort study (III)	Free flap reconstruction (*n* = 517)	Groups:	NS difference in flap failure of thrombosis rates (Bonferroni correction)	Non-randomized
			1. No anticoagulant		Higher risk given high-dose heparin
			2. 2000–3000 IU heparin, intraoperative and postop 100–400 IU 5–7 days		Different surgeons used different anticoagulant protocols
			3. 5000 IU intraoperative		Some groups underpowered
			4. 5000–10,000 IU intraop and 500–1200 IU postop		
			5. Dextran 40 25 ml/h		
Ashjian et al. 2007 [6]	Prospective cohort retrospective study (III)	*N* = 505, free flaps oncological defects	Microvascular thrombosis, partial or total flap loss, hematoma, bleeding	Flap loss (0.4% vs total flap loss (0.4%) vs 0.8%) NS	No data on thrombosis rates or flap takeback
		470 patients		Hematoma (2.3 vs 2.9%) NS	Procedures by 2 different surgeons
		1. Postoperative aspirin (A) 325 mg of 5 days (*n* = 260)			Non-randomized
		2. Postoperative LMWH (B) 5000 units until ambulant			Publication from same group of same patient series comparing outcomes with or without heparin bolus, this difference is not mentioned in this paper
Chen et al. 2006 [7]	Retrospective cohort study (III)	Free flap reconstruction (*n* = 505)	Comparing 3000 units intraoperative IV administration of heparin bolus versus no bolus	Flap failure 0.4 versus 0.8% (*p* = 0.61)	No data on returns to theater or thrombosis incidence
Nelson et al. 2014 [8]	Cohort study (III)	Hypercoagulable patients undergoing breast reconstruction (*n* = 32), personal history of VTE or thrombophilia	Heparin bolus IV, heparin infusion postop versus 5000 U sc heparin postop	Thrombosis rate 0 versus 17.6% (*p* = 0.23)	Non-randomized
					Non-contiguous
					Study of change in unit practice
Jayaprasad et al. 2013 [9]	Retrospective cohort study (III)	*N* = 172, head and neck reconstruction	Papaverine intraoperatively	Flap survival rate 95.9%	Retrospective, non-contiguous, change in practice with dextran 40 with available evidence
			0.2 ml, LMWH 5 days postop	7 postop hematomas	
			Vessels flushed with heparinized saline	No difference in failure of thrombotic complications	
			+/− dextran 40 for 5 days	Venous thrombosis 7 versus 9.3% (*p* = 0.78)	
				Arterial thrombosis 1.2 versus 3.5% (*p* = 0.62)	
Deutinger et al. 1998 [10]	Retrospective cohort study (III)	Free flap reconstruction, mainly elective (*n* = 204)	5000 IU heparin sc tid and dextran 40 bd 250 ml OR heparin 500–800 IU per hour up to 10 days Intraoperative irrigation with heparin	Flap failure 5%, dextran and heparin 7.4%, heparin 6.5% (*p* = 0.79)	Non-randomized, retrospective
Disa et al. 2003 [11]	Randomized control trial (II)	Head and neck reconstruction (*n* = 100)	Dextran for 48 h, 120 h or aspirin for \5 days	Significantly higher systemic complications in dextran groups (*p* < 0.05) No effect on flap survival	Underpowered Designed to assess systemic complications related to anticoagulation All had intraoperative 3000 IU heparin
Lighthall et al. 2011 [12]	Retrospective cohort study (III)	Head and neck reconstruction (*n* = 390)	3 groups: no prophylaxis, aspirin only (dose not given) or combination of aspirin and prophylactic dose heparin or LMWH	No significant differences in flap failure or hematoma rates between groups, more complications with aspirin than no prophylaxis	Retrospective Non-contiguous Not randomized
Sun et al. 2003 [13]	Retrospective cohort study (III)	Head and neck reconstruction (*n* = 55)	Dextran 40 20–33 cm^3^/h for 5–7 days postoperatively or no anticoagulation	Thrombosis: 4% dextran group, 0% no anticoagulant group (*p* > 0.05), 100% survival all groups No data re hematoma	Non-consecutive Underpowered Retrospective Not randomized
Riva et al. 2012 [14]	Retrospective cohort study (III)	Head and neck reconstruction (*n* = 1351)	PGE1 or dextran-40 or no antithrombotic therapy	No significant difference in flap survival/thrombosis (*p* = 0.734) No significant increase in hematomas	Retrospective Choice of treatment was based on each surgeon’s preference
Enajat et al. 2014 [15]	Retrospective cohort study (III)	DIEP/TRAM flaps for breast reconstruction, (*n* = 592)	0.6 ml nadoparine =/− 40 mg aspirin	Non-significant difference in hematoma, failure or microvascular thrombosis	Non-randomized, retrospective, different hospital/surgeons
Lee et al. 2012 [16]	Retrospective cohort study (III)	Free flaps for lower limb reconstruction (*n* = 128)	IV ketorolac 30 mg tid for first 2 postoperative days	Returns to theater 5 versus 16.7% (*p* = 0.03) No significant difference in flap failure rates	Also treated with PGE1 Non-randomized

### Quantitative analysis

Due to heterogeneity of dosing in all of these studies, lack of control studies, and combination regimes used in many studies, meta-analysis was not performed. Three studies assessed use of dextran 40; however, two of these studies included less than 25 patients treated with dextran and in one of these the dextran group 25 represented the first free flaps performed by that center.

## Discussion

This systematic review highlights the difficulty in making evidence-based treatment decisions regarding prevention of pedicle thrombosis in free flap surgery. Treatment groups varied between groups undergoing free flap breast reconstruction (*n* = 1), head and neck reconstruction (*n* = 5), lower limb reconstruction (*n* = 1), and mixed (*n* = 6). In terms of anticoagulant drug treatments, doses and durations were highly varied causing difficulty in making direct comparisons between studies. Some studies were more than 20 years old, and flap failure rates may not be comparable with current rates of failure in high-volume centers. Others have attempted to perform meta-analysis in order to assess the impact of postoperative anticoagulation; however, we felt due to lack of homogeneity between treatments that this would not be desirable [17–19]. The majority of studies lacked a negative control group instead comparing with another drug and causing difficulty in assessing the true impact of each individual intervention. In addition, the most of these studies are retrospective, and a selection bias may exist in choosing which patients receive anticoagulants with common clinical practice being to start those patients on treatment when an intraoperative difficulty or problem is encountered. In the following discussion, we consider preoperative, perioperative, and postoperative factors that may affect pedicle thrombosis risk.

### Preoperative

Senchenkov et al. describe preoperative screening for a hypercoagulable history and recommend a complex regime of aspirin, clopidogrel, dextran, and heparin based on this [20]. They describe excellent free flap success (99.5%) in a retrospective case series of 355 free flaps the majority being for breast reconstruction. Those patients who received intraoperative anticoagulation had a hematoma rate of 27%. The usefulness of this study is limited given its retrospective nature, the number of agents evaluated, the small number of patients identified preoperatively (*n* = 9), and the lack of a control arm. Nelson et al. described preoperative identification of hypercoagulable patients and use of a novel anticoagulation regime [8]. They report reduced incidence of arterial and venous thrombosis after its introduction in these high-risk patients, but the result does not reach statistical significance. These authors suggest risk stratifying patients in terms of thrombosis risk and tailoring perioperative anticoagulation dependent on risk. However, in the absence of a validated way of risk stratifying such patients, this is not straight forward and liaison with a hematologist may be beneficial when the patient is considered high risk.

There is no clear evidence that smoking increases the risk of microvascular thrombosis; however, patients should be advised to stop smoking in order to reduce their risk wound complications [21]. Tamoxifen has known prothrombotic effects and been shown to increase the risk of free flap failure when taken in the perioperative period [22]. Tamoxifen has a half-life of 2 weeks, and Mirzabeigi et al. found no increased risk of venous thromboembolism (VTE) or free flap failure in patients who ceased tamoxifen 2 weeks preoperatively [23]. As with other major surgeries, the oral contraceptive pill should be held preoperatively.

### Intraoperative

#### Surgical technique

Delicate tissue handling and meticulous microsurgical technique are key to minimizing the risk of thrombosis. Seo et al. examined ten failed free flaps histologically and found all showed evidence of thrombus formation and endothelial injury [24]. Prompt recognition of thrombus formation intraoperatively is imperative, allowing the surgeon to remove the thrombus from the vessel and analyze local factors that may have attributed to the thrombus formation (vessel size mismatch, poor-quality recipient vessels, compression/twisting of anastomosis or pedicle) before considering the need to revise the anastomosis with or without a vein graft.

Venous couplers are used routinely by some surgeons for venous anastomoses allowing better eversion of the vessel ends and reducing operative and primary ischemia time. Disadvantages include high cost, reduced experience with hand-sewn microvascular anastomosis, and they are not suitable in all cases. Kulkarni et al. found a lower rate of venous thrombosis breast reconstruction cases with their use versus sutured venous anastomosis [25].

#### Topical heparin

In rat femoral artery models, topical vessel irrigation with heparinized saline has been shown to reduce thrombosis formation and be superior to systemically administered heparin both in terms of its antithrombotic effect and risk [26–28]. This effect has not been replicated in human studies. Limitations in these animal studies include the use of prothrombotic models (for example an intimal flap or crushing the vessel ends) and the use of junior microsurgeons as operators which makes them less reflective of clinical cases.

### Postoperative

Routine use of antithrombotic drugs in microsurgery with the aim of preventing microvascular thrombosis has been reported to be as high as 96% [29]. Great overlap exists between the use of anticoagulants to prevent postoperative VTE and to prevent flap thrombosis. A recent review by Ricci et al. nicely addresses the evidence for VTE prevention in microvascular surgery. Recommendations include routine risk assessment using validated scores based on the Caprini system and use of both mechanical and chemoprophylaxis (40 mg enoxaparin or 5000 U heparin daily) based on this [30].

#### Heparin

Heparin acts by inhibiting circulating thrombin, which is an essential factor in the coagulation cascade. Thrombin converts fibrinogen to fibrin, leading to formation of cross-linked fibrin, promotes platelet aggregation, and activates coagulation factors V and VIII [31]. Disadvantages of heparin use include cost of monitoring if maintained postoperatively and an unpredictable dose response.

Animal studies have shown that heparin can reduce thrombosis formation and improve flap viability when administered topically or systemically [32, 33]. Clinical studies found no significant increase incidence of hematoma when low-dose heparin was administered at an appropriate dose for prevention of thromboembolism [5, 34]. No clinical studies demonstrated any benefit in the use of systemic heparin to prevent flap thrombosis.

#### Low molecular weight heparin

LMWH is produced by depolymerization of standard heparin into short polysaccharide fragments. LMWHs have the same inhibitory effect on activated factor X but have a weaker antithrombin activity. It is a standard prophylactic measure for venous thromboembolism for patients undergoing major surgery or those undergoing more minor surgery with risk factors for VTE. LMWH has shown varying results in animal studies in its ability to prevent thrombosis formation at microvascular anastomoses and no clear benefit in clinical studies [35, 36].

#### Dextran

Dextran, a polysaccharide, is produced by the action of the bacterium on sucrose. Dextran increases electronegative charge on platelets and endothelium therefore reducing platelet clumping. It promotes fibrin breakdown, activates plasminogen, decreases factor VIII and von Willebrand factor function therefore reducing platelet function, and acts as a volume expander. Associated side effects include pulmonary edema, renal failure, and anaphylaxis.

Ridha et al. published a study of UK plastic surgeons in 2005 and found that 45% routinely used dextran following microsurgical procedures [37]. They found no significant difference between success rates between those that did and those that did not use dextran. Dextran has largely fallen out of favor due to potential risks of anaphylaxis and pulmonary edema and the lack of an evidence base to support its use.

#### Aspirin

Aspirin acts on the cyclooxygenase enzyme in platelets reducing production of thromboxane A2, in turn preventing platelet aggregation and vasoconstriction. It also decreases the endothelial production of prostacyclin. Associated side effects include gastric ulceration and increased bleeding.

Animal studies have shown that aspirin prevents platelet aggregation and thrombus development in microvascular anastomoses [38–40]. Other studies found no effect on final patency rates [41, 42]. Enajat et al. found no difference in thrombosis or flap failure but a higher hematoma rate in patients receiving aspirin following abdominal-based free flap breast reconstruction [15].

#### Ketorolac

Ketoralac which is a reversible cyclooxygenase inhibitor reduced platelet aggregation in addition to being an effective painkiller. Shufflebarger et al. found reduced rates of microvascular thrombosis in a prothrombotic rabbit model [43]. However, in the clinical study by Lee et al., although there were reduced returns to theater in the ketorolac-treated group, there was no difference in free flap failure rates [16].

Having reviewed the available evidence, we make the evidence-based recommendations detailed in Table 2 aiming to optimize the risks of thrombosis in free tissue transfer.

**Table 2:** Evidence-based guidelines for prevention of pedicle thrombosis in free flap surgery

Preoperative	
Stop prothrombotic medications (e.g., tamoxifen, HRT)	Level 2 evidence [23]
VTE risk assessment, prescribe 40 mg enoxaparin per day, adjust dependent on weight and renal function	Level 3 evidence [30]
Liaise with hematologist for patients with known thrombotic disorders	Level 5 evidence [8]
Intraoperative	
Consider use of a venous coupler	Level 3 evidence [25]
Consider intraluminal irrigation with heparinized saline	Level 5 evidence [26–28]
Postoperative	
Heparin, aspirin, and dextran should not be routinely prescribed for free flap patients to prevent microvascular thrombosis	Level 3 evidence [5–15]

The regime proposed in this paper was based on analysis of current available evidence. With the lack of high-quality evidence, regime for optimizing the risk of thrombosis in free tissue transfer does not yet show consensus worldwide. It is widely accepted however that meticulous surgical technique is the key component to successful microsurgery. Further research in the form of high-quality randomized controlled trials is necessary to compare interventions aiming to target thrombosis.

## Conclusion

There is no clinical evidence to support the routine use of anticoagulant or antiplatelet drugs in the postoperative period, and they may increase the risk of hematoma formation. There is a lack of randomized controlled trials investigating their use in free flap surgery, and given the current low rates of flap failures, designing such a trial which is suitably powered to find a difference in flap survival between patients would require an enrollment of greater than 1000 patients and would be most feasible on a multicenter international basis. Patients with a history of thrombosis may benefit from such treatments, but further research is needed in this area including a method of stratifying their risk of microvascular thrombosis (for example, the Caprini score could be investigated as a risk assessment tool for flap thrombosis). Given these patients have a much higher risk of flap thrombosis, a randomized controlled trial may be worthwhile in this subgroup of patients.
